# RE: Positive models of suffering and psychiatry

**DOI:** 10.1192/bjb.2024.26

**Published:** 2024-06

**Authors:** Eugene G. Breen

**Affiliations:** Associate Professor of Psychiatry, Mater Misericordiae University Hospital, Dublin, Ireland. Email: ebreen@mater.ie

*The meaning of suffering*

The article by Professor Huda and the letter by Professor Prakash focus on the crucial issue of meaning in suffering. This topic could be addressed similarly by many if not all people with varying degrees of insight, lived experience and expertise. We happen to be doctors and in fact psychiatrists, so what can we bring to the table? First, I would say we have to do our jobs! We are doctors whose job is to bring healing, alleviate pain and suffering, and help people to come to terms with their illness and its impact on their lives and possibly what it means to them. To focus on meaning in suffering may be more appropriate for close friends, family or pastors. A person's world view, belief system and social support network largely determine what they consider to be the meaning, cause or message of illness. Psychiatrists who prioritise the meaning aspect and do not treat the illness may not be providing the service they are qualified for and paid to do. Undoubtedly, understanding why suffering is happening can mitigate anguish, confusion and resentment (to mention a few reactions), and as such, insofar as it is within our job spec, we should facilitate this. However, referral to a pastor, friend or confidante may be more appropriate. The humanity, compassion and empathy, and clinical professional skill of the doctor may be our best combination to bring healing to a suffering person. To overfocus on the supportive, meaningful side to the neglect of the doctor's role in curing and treating illness would be detrimental to our profession. First, we are psychiatrists with a definite job spec; then, we are humans with empathy and compassion and expertise to support our curing role; and, finally, we are learners deepening our own grasp of suffering and its causes and cures from the example of our patients. I congratulate both authors and also strive to tease out where the golden mean lies in this very human drama.

